# Base J and H3.V Regulate Transcriptional Termination in *Trypanosoma brucei*

**DOI:** 10.1371/journal.pgen.1005762

**Published:** 2016-01-21

**Authors:** Danae Schulz, Maryam Zaringhalam, F. Nina Papavasiliou, Hee-Sook Kim

**Affiliations:** Laboratory of Lymphocyte Biology, The Rockefeller University, New York, New York, United States of America; Universidade de Lisboa Instituto de Medicina Molecular, PORTUGAL

## Abstract

*Trypanosoma brucei* is a protozoan parasite that lacks many transcription factors found in other eukaryotes, such as those whose binding demarcates enhancers. *T*. *brucei* retains histone variants and modifications, however, and it is hypothesized that it relies on epigenetic marks to define transcription-related boundaries. The histone H3 variant (H3.V) and an alternate nucleotide, base J (ß-D-glucosyl-hydroxymethyluracil), are two chromatin marks found at both transcription termination sites (TTSs) and telomeres. Here, we report that the absence of both base J and H3.V result in transcription readthrough and the appearance of antisense transcripts near TTSs. Additionally, we find that maintaining the transcriptional silencing of pol I-transcribed telomeric Variant Surface Glycoprotein (*VSG*) genes appears to be dependent on deposition of H3.V alone. Our study reveals that gene expression depends on different epigenetic cues depending on chromosomal location and on the transcribing polymerase. This work provides insight into how these signals may have evolved into the more nuanced and fine-tuned gene regulatory mechanisms observed in other model systems.

## Author Summary

In eukaryotes, DNA is wrapped around histones to form chromatin. Modifications on the DNA itself, or on the canonical histones around which the DNA is wrapped, can lead to changes in gene expression. Alternate histones are also used to regulate gene expression. The African trypanosome, the causative agent of African sleeping sickness, transcribes its genes in long arrays called polycistronic transcription units (PTUs). In this study, we describe how the African Trypanosome uses two chromatin marks to regulate genes that lie close to the end of the PTU or close to the ends of chromosomes, called telomeres. One mark, base J, is on the DNA itself, while the other utilized mark is an alternate histone protein called H3.V. In the absence of these marks, there is an increase in antisense RNA that corresponds to genes that lie near the ends of the PTUs, and an increase in sense RNA for genes near telomeres. Since trypanosomes are evolutionarily distant from humans, these findings shed light on how gene expression mechanisms that are modulated by chromatin marks may have evolved to produce the complex gene regulatory networks found in our own tissues.

## Introduction

*Trypanosoma brucei*, a eukaryotic parasite that causes African sleeping sickness, belongs to the order Kinetoplastida and diverged from mammals ~500 million years ago. *T*. *brucei* is heteroxenous, requiring two obligatory hosts to complete its life cycle: a mammalian host and an insect host (*Glossina spp*, the tsetse). Trypanosomes proliferate in the tsetse gut as procyclic forms (PF) [1] and differentiate into non-proliferative metacyclic forms in tsetse salivary glands. After *T*. *brucei* infects a mammalian host, it differentiates into the bloodstream form (BF) and proliferates in the host’s extracellular spaces. The cycle is completed when BF *T*. *brucei* reaches a quiescent state and gets ingested by a tsetse.

*T*. *brucei* branched early in eukaryotic evolution, and differs substantially from other eukaryotes in its regulation of gene expression. It is thus of great evolutionary interest. One prominent difference concerns transcription of protein-coding genes by RNA polymerase II (pol II), which diverges in two important ways from other well-studied eukaryotes. First, transcription occurs polycistronically, and most genes are arranged in polycistronic transcription units (PTUs). Mature mRNAs are then generated post-transcriptionally by coupled *trans*-splicing and polyadenylation reactions that trans-splice a 39-nucleotide leader sequence onto every mRNA [2]. Second, some general transcription factors and one pol I-specific factor have been identified in *T*. *brucei* [3–5], but the parasite lacks many of the sequence-specific transcription factors found in other eukaryotes that bind to *cis*-regulatory sequences, and *cis*-regulatory sequences themselves have not been well characterized. The lack of these regulatory factors, and the fact that histone modifications and histone variants are deposited at sites of putative transcription initiation and termination, has led to the idea that chromatin marks are important for demarcating PTUs [6]. Prior experiments using chromatin immunoprecipitation and sequencing (ChIP-seq) have correlated the presence of certain histone modifications and histone variants with transcription initiation sites (H3K4me^3^, H4K10ac, and H2 variants H2.AZ and H2.BV at transcription start sites (TSSs)) or transcription termination sites (H3.V and H4.V at transcription termination sites (TTSs)) [6,7]. However a mechanistic involvement of these marks with transcriptional regulation has not been demonstrated.

In addition to these distinct chromatin features, pol II transcription initiation and termination regions contain a kinetoplastid-specific DNA modification, known as base J (ß-D-glucosyl-hydroxymethyluracil) [8]. It arises via two steps that modify dT: the J-Binding Protein-1 (JBP1) and JBP2 (homologs of the mammalian TET proteins) mediated hydroxylation of thymidine to generate a hydroxymethyl-dU (hmU) intermediate [9], followed by the glucosylation of hmU by the J-associated Glucosyl Transferase (JGT) [10]. Base J synthesis requires both JBP1/2 and JGT [10,11]. Base J is enriched at PTU flanks, where it coincides with, but does not depend on, the presence of H3.V [12]. It has recently been demonstrated that depletion of base J in *Leishmania* results in readthrough transcription at sites of transcription termination [13,14], while deletion of H3.V does not have this phenotype [15]. In contrast, depletion of base J in *T*. *brucei* does not cause readthrough at convergent strand switch regions, though it does affect termination at specific sites proximal to J deposition [13]. Deletion of H3.V has not been characterized with respect to transcriptional readthrough in *T*. *brucei*. Based on these results, we hypothesized that while base J may be the dominant mark for transcription termination in *L*. *major*, H3.V may compensate for the lack of base J in *T*. *brucei*, and its role in the epigenetic network of *T*. *brucei* may differ from that of *L*. *major*. We surmised that the effects of base J and H3.V in *T*. *brucei* might best be assessed by removing them simultaneously, and sought to ask whether perturbation of chromatin marks at sites of transcription termination would have any functional consequence on gene expression.

In addition to TTSs, J and H3.V (but not H4.V) marks coincide over telomeric regions in *T*. *brucei*. Variant Surface Glycoprotein (*VSG*) genes, encoding the major *T*. *brucei* surface antigen, are frequently found near telomeres. One genomic location for telomere-proximal *VSG*s is the Bloodstream-form Expression Site (BES), which contains a polycistronic transcription unit that is transcribed by RNA pol I. Fifteen BESs have been found in the Lister 427 strain [16], and each BES terminates at the telomere with a telomere-proximal *VSG*. Another class of telomere-proximal *VSG*s is found in metacyclic expression sites (m*VSG*s). These sites are also transcribed by pol I and m*VSG* is expressed during the metacyclic stage in the tsetse salivary gland. Minichromosomes have also been found to contain *VSG* genes, but these sites lack obvious pol I promoter sequences. A large number of other *VSG* genes are scattered throughout the genome, but not all of their genomic locations are known [17]. In BF cells, only one BES is transcriptionally active at any given time, ensuring the monoallelic expression of a single coat protein that is essential to immune evasion and, therefore, survival of the organism in the mammalian host. Given the colocalization of base J and H3.V at telomeric sites, we sought to test whether the combination of J and H3.V marks are necessary to maintain silencing of *VSG* expression units in BF trypanosomes, taking advantage of the telomeric location of *VSG* genes to interrogate telomeric silencing in general.

Using high-throughput, strand-specific sequencing of poly-A+ mRNA (RNA-seq) on genetic mutants that lack H3.V, base J, or both marks, we demonstrate that simultaneous deletion of base J and H3.V increases the amount of antisense transcripts for genes proximal to transcription termination sites, where both marks are found. We also demonstrate that of the two marks, H3.V appears to be more dominant than base J with respect to maintaining silencing of telomeric *VSG* genes. Thus our experiments suggest that perturbations of the chromatin landscape in different regions of the genome (transcription termination sites *vs*. telomeres) have functional effects on gene expression, and that the dependence on a particular chromatin mark differs depending on chromosomal location and/or specific features of the transcribing polymerase (RNA pol II *vs*. pol I).

## Results

### Simultaneous deletion of base J and H3.V results in viable cells

To study the roles of base J and H3.V, we generated knockout cell lines in BF *T*. *brucei* using previously published methods [18]. To make BF trypanosomes deficient in base J, we first removed both alleles of *JBP1*, then both alleles of *JBP2*. In both cases, we used deletion-cassettes containing hygromycin- or puromycin-resistance genes fused to *Herpes simplex* virus thymidine kinase (*HYG-TK* or *PUR-TK*) and flanked by loxP sites, allowing the markers to be removed by transient expression of Cre-recombinase and reused. *JBP1Δ/Δ JBP2Δ/Δ* double mutants, which we will henceforth refer to as *JΔ* mutants, are completely null for base J [11]. To generate BF trypanosomes deficient in only H3.V or both H3.V and base J, respectively, we sequentially deleted both alleles of *H3*.*V* either from a WT or *JΔ* background using the deletion cassette *H3*.*VΔPUR* (pJEL76) and *H3*.*VΔHYG* (pJEL38) [19].

**Fig 1:**
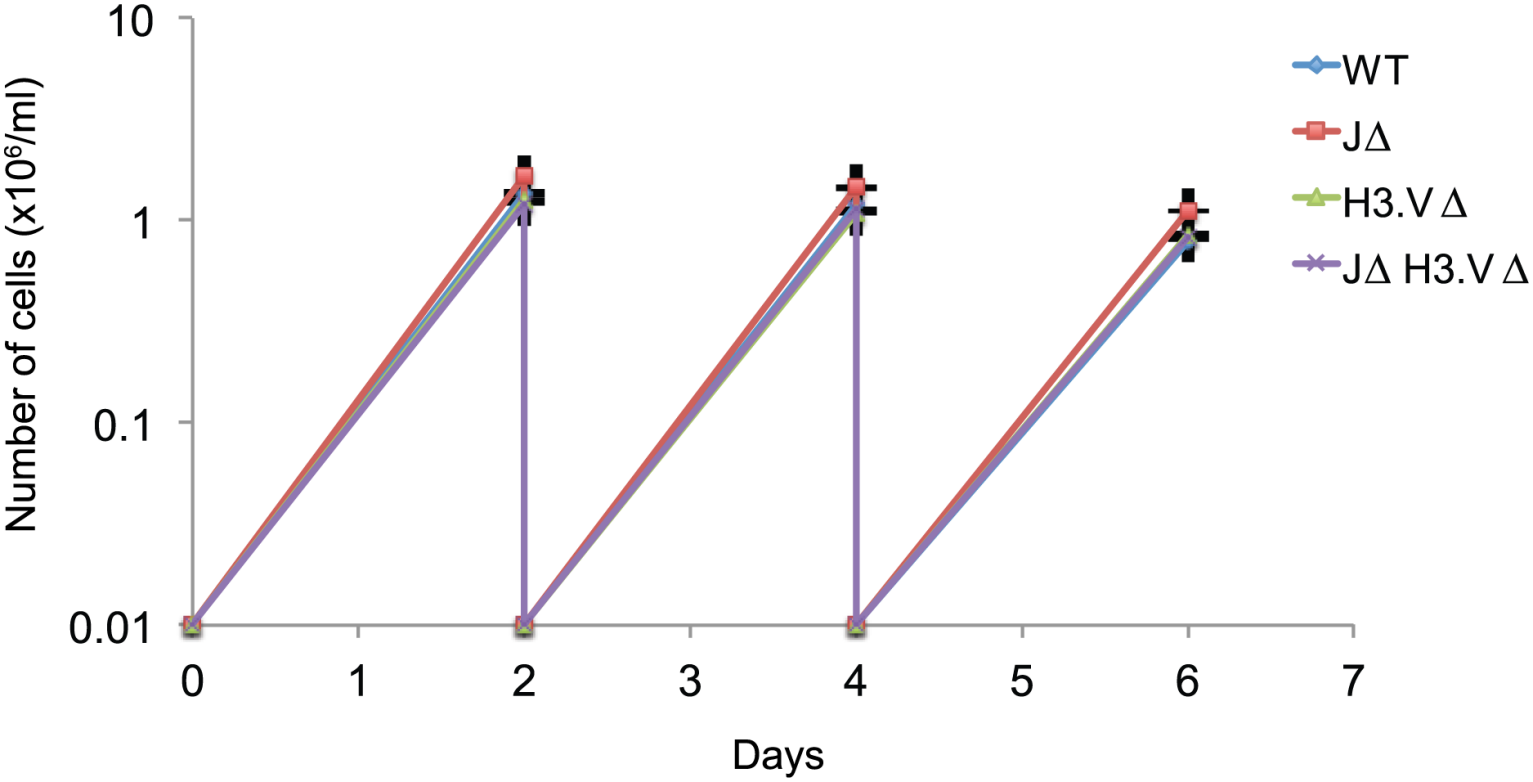
Cells that are simultaneously deleted for base J and H3.V are viable. WT, *JΔ*, *H3*.*VΔ*, and *JΔ H3*.*VΔ* cells were diluted to 10,000 cells/ml and cells were counted after two days of incubation. This was repeated two more times. Cells were counted three times. Error bars are shown and there were no significant differences between WT and mutant cells.

H3.V and base J are not synthetic lethal, as *JΔ H3*.*VΔ* trypanosomes were viable. Growth curves revealed that there was no obvious defect in growth for any of the mutants (Fig 1) and there were no obvious morphological differences.

### H3.V is important for maintaining silencing of a subset of *VSG* genes

**Fig 2:**
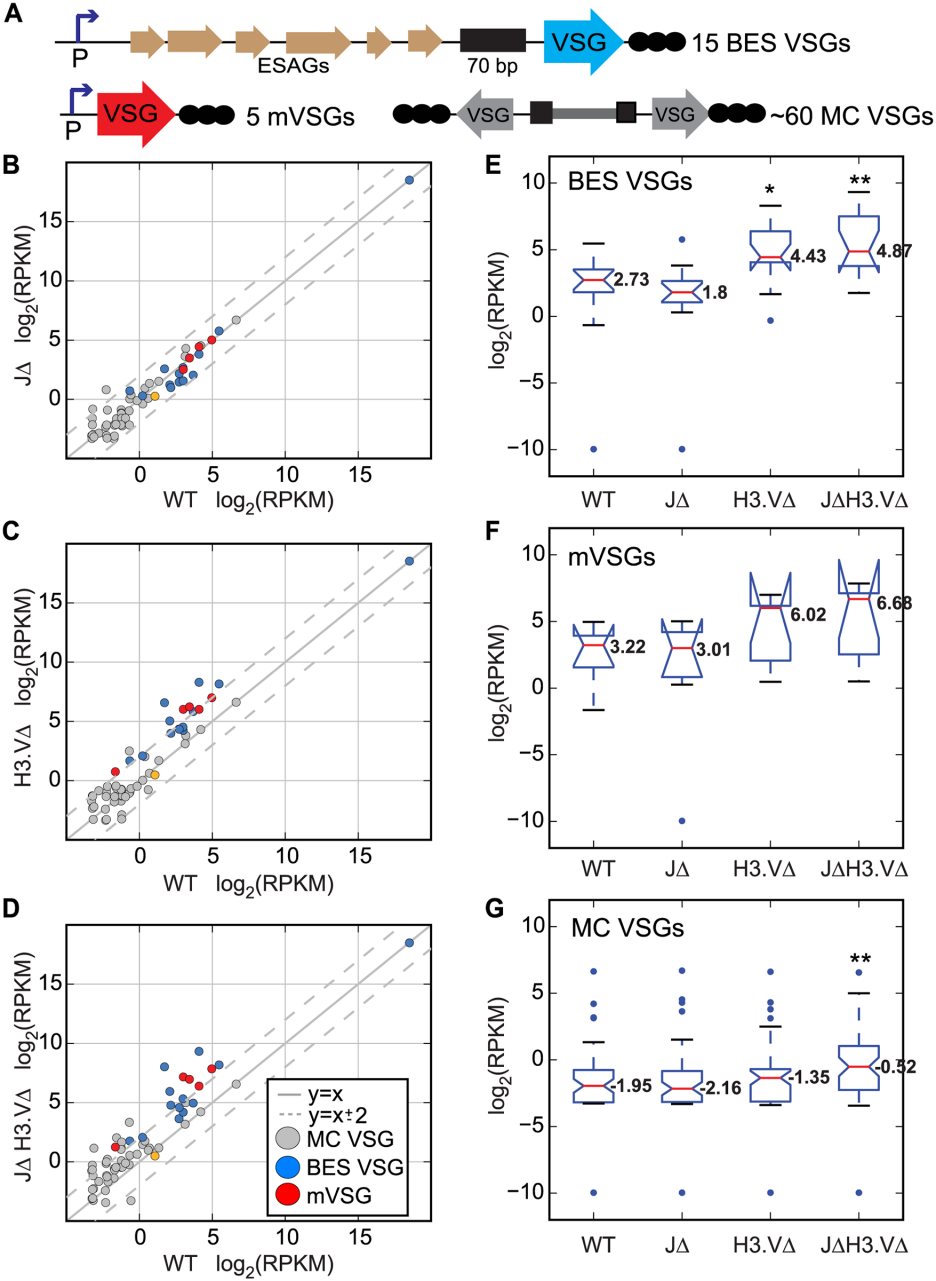
H3.V is important for maintaining silencing of a subset of *VSG* genes. (A) Schematic diagrams of *VSG* locations; BES *VSG* (Bloodstream-form Expression Site *VSG*), m*VSG* (metacyclic *VSG*), and MC *VSG*, (minichromosomal *VSG*). Reads from the RNA-seq experiment were aligned to the *VSG*nome database and log_2_(RPKM) values were obtained for all *VSG* genes. (B-D) log_2_(RPKM) values for *VSG*s with known chromosomal locations were compared between the indicated mutant samples and WT in scatter plots. Dotted grey lines indicate changes that are 4-fold up or down. Blue dots are BES-*VSG*s, red are metacyclic promoter-associated *VSG*s (m*VSG*) and grey are minichromosomal *VSG*s. The orange circle corresponds to *VSG* 582, which is associated with a metacyclic promoter in an atypical metacyclic ES located far upstream of the telomere [22]. MC, minichromosomal, m, metacylic promoter-associated. *ESAG*, Expression Site Associated Gene. (E-G) Notched boxplots of log_2_(RPKM) values were generated for all *VSG*s located in BESs (E), metacyclic expression sites (F), or minichromosomes (G). Expression values for the *VSG* in the active BES are not shown on the BES boxplots in (E) but do not vary significantly between genotypes, ranging from 18.47–18.54. Red lines indicate median values, and are indicated with numbers on the plot. Upper and lower blue lines mark the interquartile range. Whiskers mark data falling within the 1^st^ and 4^th^ quartiles. Outliers are shown as blue dots. Notches are calculated as ±1.58 x IQR/√n where n indicates the number of samples and IQR is the inner quartile range. Significant differences are demarcated by * (*P* < 0.05) and ** (*P* < 0.01) between indicated mutant and wild-type values as measured by a Mann-Whitney U statistical test.

Both base J and H3.V are highly enriched at telomeres in BF cells [19–21]. As some *VSG* genes are located near telomeres (Fig 2A, diagram, BES *VSG*s or m*VSG*), we asked whether base J and H3.V are important for maintaining silencing of inactive telomeric *VSG*s by assessing expression levels of these telomeric *VSG*s in cells lacking one or both chromatin marks. We also assessed expression levels for minichromosomal *VSG*s (MC *VSG*s), which are assumed to be proximal to telomeres. We isolated polyA+ RNA from each of the mutants described above, as well as from the parental cell line (referred to as WT). Libraries for high-throughput sequencing were generated using 3 independent cultures from each genotype (for a total of 12). Reads from each library were uniquely aligned allowing for two mismatches to the megabase chromosomes. Unmapped reads were then uniquely aligned allowing for two mismatches to the *VSG*nome [17]. A log_2_(RPKM) (Reads Per Kilobase Per Million mapped reads) value was generated for reads aligning to each *VSG* and these values were averaged across the 3 replicates. We then compared WT and mutant log_2_(RPKM) values for *VSG*s with known subtelomeric locations in Lister427 cells (BESs, minichromosomes) as well as those known to be associated with metacyclic promoters [17,22] (Fig 2B–2D). We also generated notched boxplots for log_2_(RPKM) values of BES, metacyclic, and minichromosomal *VSG*s to elucidate the overall distribution of the data (Fig 2E–2G, respectively).

In the *H3*.*VΔ* mutant, 40% (6 of 15) of BES-associated *VSG*s, and 4 of the 5 metacyclic *VSG*s were >4-fold upregulated (Fig 2C, S1 Table). The median log_2_(RPKM) values for BES and metacyclic *VSG*s were higher in the *H3*.*VΔ* mutant and the distribution of log_2_(RPKM) values was shifted upward (Fig 2E and 2F). This effect was recapitulated in the *JΔ H3*.*VΔ* mutant (Fig 2D), with 7 BES-associated and 5 metacyclic *VSG*s >4-fold upregulated in these cells (S1 Table). Median log_2_(RPKM) values were also higher in the *JΔ H3*.*VΔ* mutant (Fig 2E and 2F). A Mann-Whitney U test was performed between WT and mutant cells to address whether the change in median values was significant. This test revealed that the difference in *VSG* expression at BES *VSG*s in both the *H3*.*VΔ* mutant and the *JΔ H3*.*VΔ* mutant were significant. *P* values for these tests are provided in S2 Table. While the majority of individual metacyclic *VSG*s are upregulated in the *H3*.*VΔ* and *JΔ H3*.*VΔ* mutants, the difference in gene expression for this group of genes was not statistically significant according to our statistical test. We used antibodies against VSG3 (which is located at a BES but transcriptionally silenced in our strains) and detected its presence by western blot analysis (S1 Fig), thus further validating BES-associated *VSG* derepression. On the other hand, in the absence of base J alone, no BES or metacyclic promoter associated *VSG*s were >4-fold upregulated (Fig 2B, S1 Table). We conclude that the *H3*.*V* mark is important for maintaining silencing of BES *VSG*s, while base J appears dispensable for silencing of these *VSG*s.

Unlike the BES and metacyclic *VSG*s, transcripts of minichromosomal *VSG*s (which lack canonical pol I promoters) were upregulated in the absence of both base J and H3.V. Of 56 MC *VSG*s, 13 minichromosomal *VSG*s showed >4-fold upregulation in the *JΔ H3*.*VΔ* mutant (Fig 2D), while 2 and 1 minichromosomal *VSG*s were >4-fold upregulated in the *JΔ* and *H3*.*VΔ* strains respectively (Fig 2B and 2C, S1 Table). Boxplots also show an increase in the median log_2_(RPKM) values in the *JΔ H3*.*VΔ* mutant (Fig 2G), which were statistically significant. The increase in expression of subtelomeric *VSG*s by deleting *H3*.*V* alone suggests that H3.V is the dominant epigenetic mark for telomeric silencing. The fact that more minichromosomal *VSG*s become upregulated when base J is also absent may indicate that base J is able to partially compensate for the lack of H3.V at these genomic locations. The absence of canonical pol I promoters from minichromosomes would imply that the upregulation could be due to promiscuous transcription by pol II or unknown factors.

When we applied RNA-seq analysis to ~2,500 annotated *VSG* sequences [17], we found that transcription of 45 and 57 *VSG* genes were >4-fold upregulated in the absence of base J or H3.V, respectively (S2A and S2B Fig, S1 Table). A more pronounced effect was observed in *JΔ H3*.*VΔ* cells, where 193 *VSG* genes were >4-fold upregulated (S2C Fig, S1 Table), representing about 7.5% of the annotated *VSG*s. Median log_2_(RPKM) values increased slightly in the absence of base J or H3.V, with the greatest difference found in the *JΔ H3*.*VΔ* mutant (S2D Fig). Statistical testing indicated that differences in transcript levels between WT and mutant cells was significant in each of the mutant strains tested (*JΔ*, *H3*.*VΔ*, *and JΔ H3*.*VΔ*) (S2 Table). All *VSG* raw read counts are reported in S1 Data.

Combined with the analysis of *VSG*s at known chromosomal locations described above, these results highlight a role for both base J and H3.V in maintaining a chromatin structure that promotes silencing of a subset of *VSG* genes. The additive effect of removing base J and H3.V on transcription of the set of *VSG*s with uncharacterized locations suggests that collaborative reading of these chromatin marks may be more common in regions further from the telomeres. The effect observed upon removal of both marks implies that the factors responsible for reading these marks and maintaining silencing may operate in different pathways that have some functional redundancy [23]. Overall, it appears that the role for certain chromatin marks (and by extension chromatin structure) in maintaining silencing of *VSG* genes appears to differ depending on chromosomal context.

Increased *VSG* levels could indicate either derepression of silent *VSG*s or an increase in *VSG* switching rates, possibly due to effects on telomere length [24]. However, the *VSG* switching rate was not significantly elevated in *H3*.*VΔ* or *JΔ* mutant cells, and was only slightly elevated in *JΔ H3*.*VΔ* cells (S3A Fig). In mammals, telomere shortening led to reduction of repressive epigenetic marks such as H3K9me^3^ and H4K20me^3^ [25]. As base J and H3.V are both located at telomeres in *T*. *brucei*, we examined telomere length in the *H3*.*VΔ* and *JΔ* mutants but did not find differences between the genotypes either in speed of lengthening or in maintenance (S3B–S3E Fig).

### Transcript levels at sites of transcription termination increases following deletion of base J and H3.V

Regions flanking PTUs can be binned into three distinct types. Sites where both PTUs initiate but run in opposite directions are putative sites of transcription initiation, termed divergent strand switch regions (dSSRs). Sites where both PTUs terminate coming from opposite directions are putative sites of transcription termination, termed convergent strand switch regions (cSSRs). Sites where one PTU ends and another starts are referred to as head-to-tail or HT (S4 Fig). Previous studies indicated that pharmacologic depletion of base J results in increased gene expression for genes downstream of specific sites of base J deposition in *T*. *brucei* [13], but did not find consistent evidence for readthrough transcription at cSSRs, indicating that an additional mark might help maintain termination signals in the absence of base J at cSSRs. Both base J and H3.V are found at cSSRs in *T*. *brucei* (S4 Fig), and it is hypothesized that these marks might act as signals for transcription termination. We thus wondered whether the absence of both base J and H3.V might result in an increase in the number of transcripts found within cSSRs.

**Fig 3:**
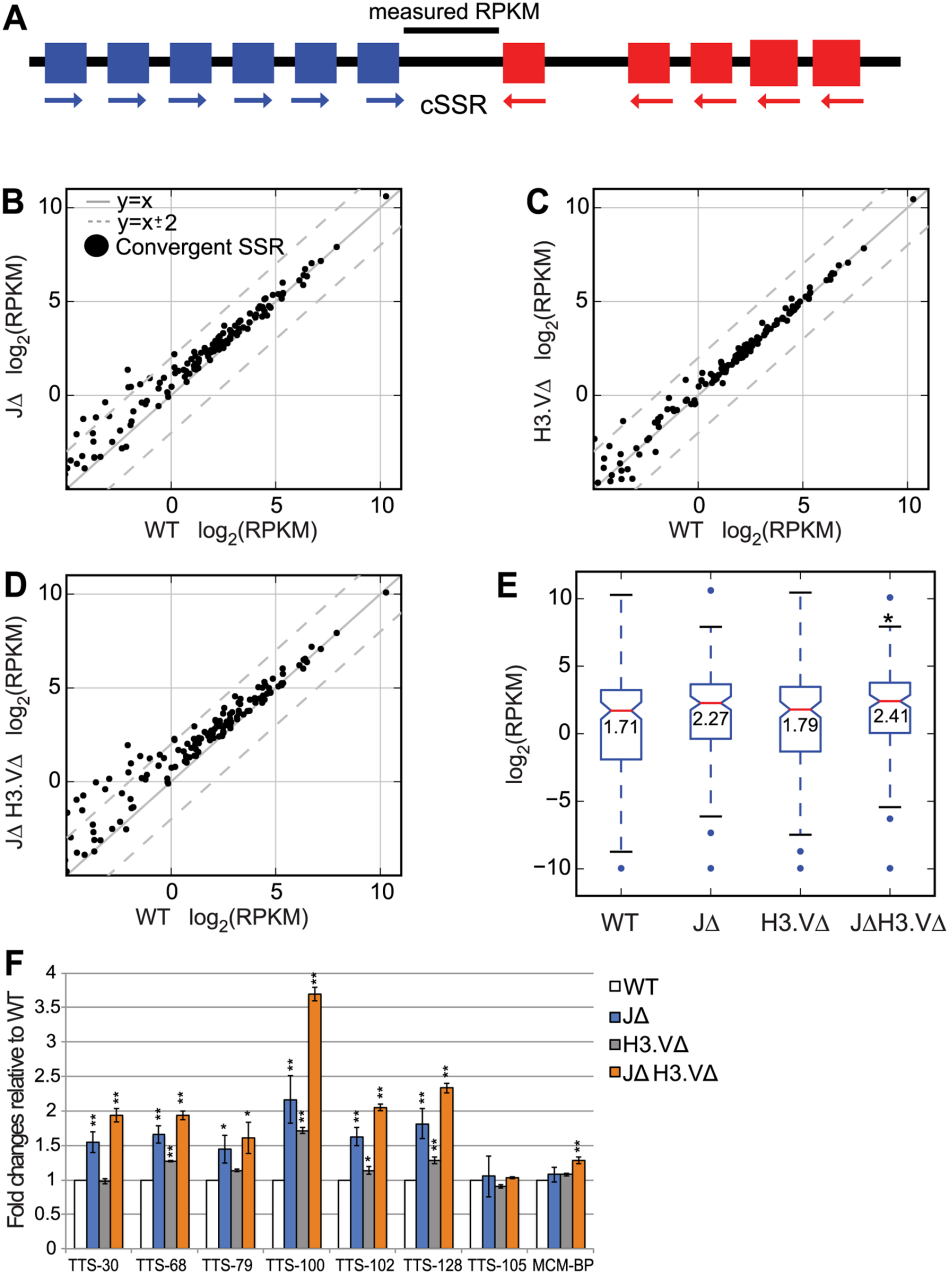
The absence of base J and H3.V results in an increase in the number of transcripts between genes that flank convergent SSRs. (A) Schematic, the log_2_(RPKM) value is computed only for the region lying between the two genes that flank the cSSR. (B-D) Scatter plots showing log_2_(RPKM) values for WT and indicated mutant cells computed for regions between genes that flank the cSSR, defined as between the 3´ end of the last gene in the (+) strand PTU and the 5´ end of the first gene in the (-) strand PTU. Dotted grey lines indicate changes that are 4-fold up or down. Comparisons between WT and *JΔ* cells are shown in (B), WT and *H3*.*VΔ* cells in (C), and WT and *JΔ H3*.*VΔ* cells in (D). (E) Boxplot for log_2_(RPKM) values computed for the regions plotted in B-D. Boxplots are displayed as in Fig 2G. * indicates a significant difference (*P* < 0.05) as measured by a Mann-Whitney U test. ** *P* < 0.01 (F) q-PCR experiment on total RNA in WT and mutant cells to determine transcripts level at selected TTSs. TTS-30, 68, 79, 100, 102, and 128 all showed higher expression of polyA+ RNA in *JΔ H3*.*VΔ* cells when compared to WT cells in the RNA-seq experiment. TTS-105 did not show upregulation in the RNA-seq dataset and was used as a negative control. MCM-BP primers were used as an additional negative control. Significance is measured using a Student’s T test.

Stranded RNA-seq libraries were prepared from 3 independent cultures from each genotype, and sequencing reads were aligned uniquely to the megabase chromosomes allowing for 2 mismatches. We investigated polyA+ transcript levels extending past the coding regions of the genes that flank cSSRs. We obtained polyA+ reads that mapped to regions between the coding regions of genes that flank convergent PTUs (Fig 3A), which we will refer to as convergent strand switch regions (or cSSRs for simplicity). We defined this region as starting 1 bp past the coding region of the (+) strand gene at the end of one PTU and ending 1 bp before the start of the (-) strand gene at the end of the second PTU (Fig 3A). log_2_(RPKM) values were generated for each of these regions and the three values for each replicate within one genotype were averaged. Polyadenylation sites have previously been shown to vary quite widely both within a gene and across different genes, and can extend up to 6,000 nucleotides past the end of the coding region of the gene, with the median length estimated at ~400 bp [26]. Overall, we found an increase in polyA+ transcripts specifically in cSSR regions in the *JΔ H3*.*VΔ* mutant (Fig 3B–3E). Median log_2_(RPKM) levels and inner quartile ranges for these regions were also increased in each of the mutants, and this increase was significant in *JΔ H3*.*VΔ* cells according to a Mann-Whitney U test (Fig 3E, S4 Table). We were able to confirm an increase in total transcript levels within these regions by performing q-PCR on selected sites (Fig 3F, TTS-105 and MCM-BP did not show increased log_2_(RPKM) values in the RNA-seq dataset and are used here as negative controls).

We analyzed the number of cSSRs with a significant increase in gene expression in each of our mutant lines by performing statistical tests on each set of replicates using Benjamimi and Hochberg correction. We found that 60% of all cSSR sites showed a significant change in log_2_(RPKM) values in the *JΔ H3*.*VΔ* mutant, while 42% and 7% of these sites showed significant changes in log_2_(RPKM) values in the *JΔ* and *H3*.*VΔ* mutants, respectively (S3 Table).

We also performed an analysis of head-to-tail sites. H4K10ac marks have previously been shown at both divergent strand switch regions (dSSRs) and at head-to-tail sites, and these regions are also bound by Bdf3 [6]. We defined head-to-tail sites as regions that are bound by Bdf3 that lie within the middle of a PTU (as opposed to a dSSR). When we analyzed these regions by stranded RNA-seq, we found that there was a significant increase, as measured by a Mann-Whitney U test, in both sense and antisense reads at these regions in *JΔH3*.*VΔ* mutant cells, but that the effect was more pronounced in antisense transcripts (S5 Fig and S5 Table). Taken together, these results led us to speculate that the absence of both base J and H3.V might result in transcriptional readthrough at regions of transcriptional termination.

### The absence of base J and H3.V results in transcription readthrough at sites of transcription termination

**Fig 4:**
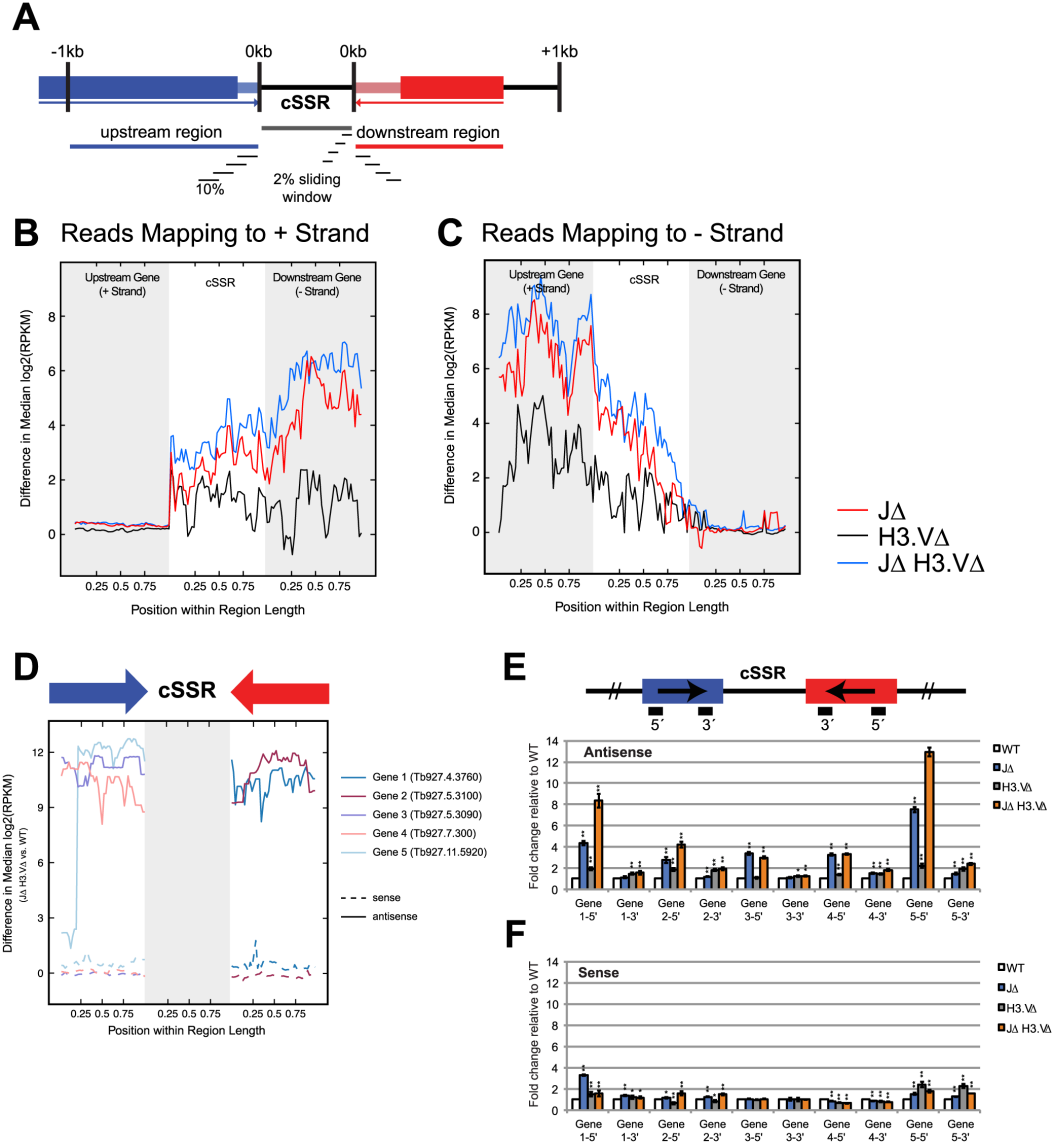
Base J and H3.V are required for proper transcription termination at cSSRs. (A) Diagram of windows used to compute values. Here, we defined the cSSR as a 1 to 5 kb long region between the 3´ UTRs of the last genes of converging PTUs. 3´ UTRs are depicted as thinner, lighter boxes. The regions 1 kb upstream and downstream of a given cSSR of interest (up to 1kb in length) and the cSSR itself were divided into 10% sliding intervals with a step size of 2% to ensure an equal number of intervals for comparison. If the cSSR flanking gene is less than 1 kb long, only the region spanning the full length of the gene was used to calculate sliding windows. (B, C) Median difference in log_2_(RPKM) values were calculated between WT and mutant cells for sliding windows in each cSSR and cSSR proximal region. The mean difference for each window across all cSSRs and cSSR proximal regions was then calculated. Mean difference in expression for transcripts mapping to the positive strand (B) or negative strand (C) was plotted against the intervals along the cSSR and cSSR proximal region. (D) Median difference in log_2_(RPKM) values between WT and *JΔ H3*.*VΔ* for a representative set of five genes. Median differences were calculated for sense (dashed) and antisense (solid) transcripts. Arrows above denote the direction of transcription. Genes 1 through 5 are Tb927.4.3760, Tb927.5.3100, Tb927.5.3090, Tb927.7.300 and Tb927.11.5920 respectively. (E-F) Stranded q-PCR results for genes in (D). q-PCR was performed with probes specific to the 5´ and 3´ ends of each gene, according to schematic shown in (E). Antisense (E) and sense (F) transcript abundance with respect to WT is shown for each mutant. Random hexamer-primed *TbURA3* was used for normalization. * indicates *P* < 0.05 and ** *P* < 0.01.

To explore the idea that readthrough takes place at regions of transcription termination, we wanted to ask whether the absence of base J and H3.V might result in polyA+ reads that extend past the 3´ UTRs of genes immediately proximal to cSSRs. We interrogated the set of cSSR proximal genes for which 3´ UTR information was defined [26]. Because 3´ UTR length is highly variable, we used the maximum 3´ UTR length defined for each of these genes [26]. We then restricted our analysis to the set of cSSRs whose boundaries were delimited by defined 3´ UTRs and whose length was between 1,000 and 5,000 bp. To determine whether increased transcripts within the cSSRs result from readthrough of cSSR proximal genes, we computed the average difference in coverage between WT and mutant cells for sliding windows starting from up to 1 kb upstream of the 5´ end of the cSSR and ending 1 kb downstream of the 3´ end of the cSSR (Fig 4A). Sliding windows were calculated by dividing the length of each region (upstream 1kb, the cSSR itself, and downstream 1kb) into 10% intervals and sliding these intervals by increments of 2%. Because cSSR and cSSR proximal region lengths vary, intervals were computed using percentages rather than length to ensure an equal number of windows for comparison across cSSRs. In this analysis, we found that the greatest difference in transcript levels between WT and *JΔ H3*.*VΔ* mutant cells is found immediately following the end of the last 3´ UTR for the last gene at the end of the PTU (Fig 4B and 4C). That is, if a PTU is found on the (+) strand, the difference in transcript levels for WT and *JΔ H3*.*VΔ* mutant cells for the last gene in the PTU remain minimal until the end of the 3´ UTR. More transcripts are then observed in the *JΔ H3*.*VΔ* mutant cells when compared to WT cells in cSSR regions, and that difference increases crossing into the opposite PTU. While antisense transcription does persist into the cSSR past the 3´ UTR in WT cells, the abundance of antisense transcripts approaches 0 moving into the opposite PTU. Conversely, an appreciable level of antisense transcripts persists through the cSSR, extending further into the opposite PTU. Consequently, a more pronounced difference in antisense transcript levels within cSSR proximal regions is observed.

To more directly measure the level of antisense transcription in cSSR proximal genes, we performed stranded q-PCR on five candidate genes that showed a marked difference in antisense transcript levels between WT and *JΔ H3*.*VΔ* (Fig 4D–4F). q-PCR analysis confirmed that more antisense transcripts were produced in the absence of base J and H3.V with respect to WT (Fig 4E). Conversely, the difference in sense transcripts in *JΔ H3*.*VΔ* compared to WT was much lower than for the antisense transcripts (Fig 4F). Together, these data suggest that the absence of base J and H3.V may cause a perturbation at the site of transcription termination.

### The absence of base J and H3.V causes transcriptional readthrough, resulting in antisense transcripts for genes in the neighboring PTU

**Fig 5:**
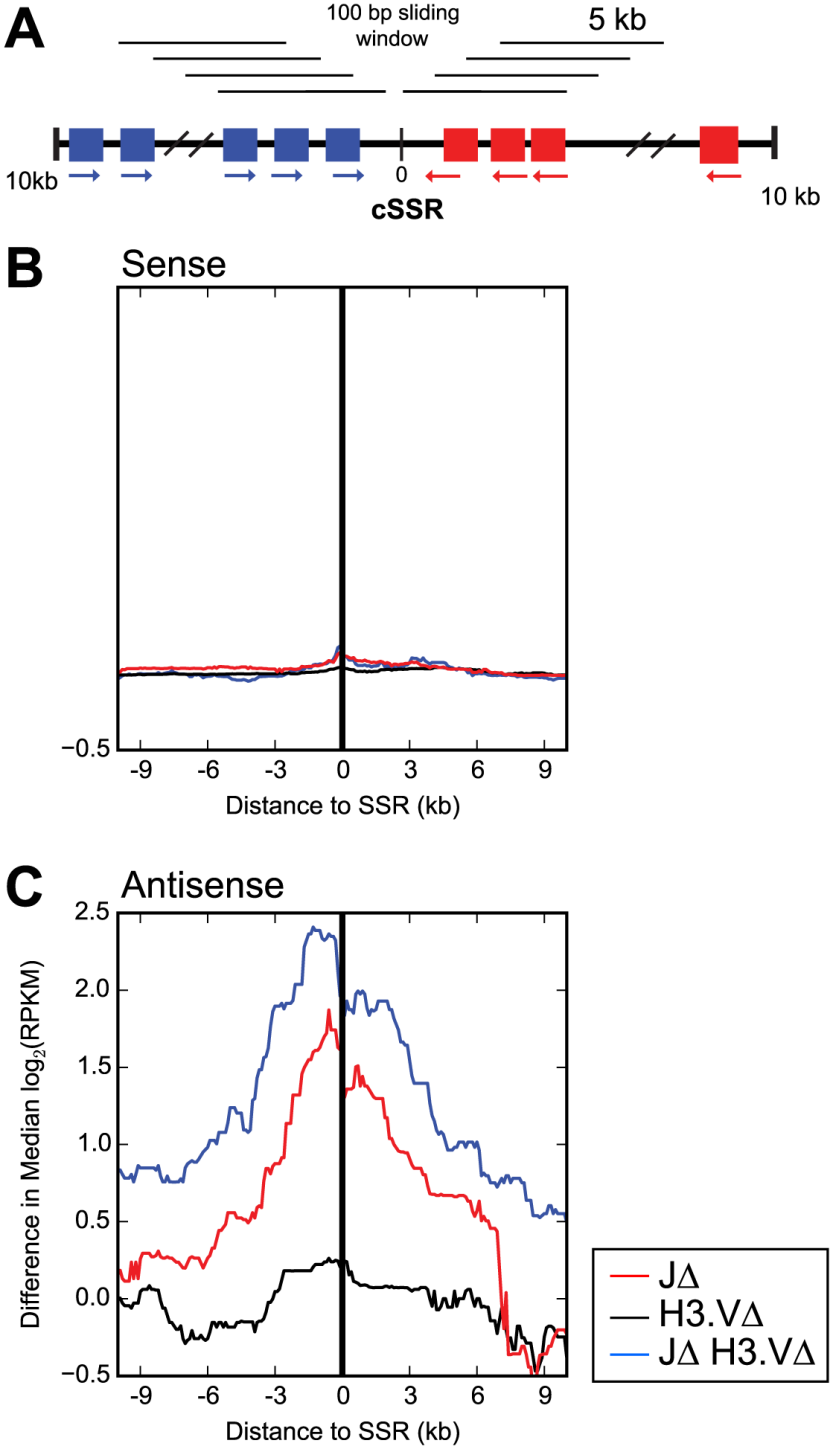
Deletion of base J and H3.V results in the production of antisense transcripts near regions of transcription termination. (A) Diagram of windows used to compute values. (B) and (C) The median difference between the indicated mutant cell log_2_(RPKM) value and the WT log_2_(RPKM) value was calculated for all genes within 5 kb sliding windows with step size 100bp extending from the halfway point of the region lying between the 3´ end of the last gene in the (+) strand PTU or the 5´ end of the first gene in the (-) strand PTU that flanks the cSSR. Median difference in gene expression was plotted against the distance of each 5 kb window from the halfway point. (B) Sense transcripts. (C) Antisense transcripts. The halfway point is indicated by a black line with 0 in the diagram in (A).

Readthrough transcription proceeding from one PTU into the converging PTU produces antisense transcripts. Polyadenylated antisense transcripts have previously been identified in *T*. *brucei* [26], so we next asked whether antisense transcripts could be detected in our stranded polyA+ RNA-seq libraries. Indeed, we were able to identify antisense transcripts and furthermore found that antisense transcripts are more prevalent in base J and H3.V mutant cells. We divided the regions upstream and downstream of each cSSR into sliding windows of 5,000 bp, with a step size of 100bp, and then calculated the median difference in log_2_(RPKM) values between the WT and mutant cells for all the genes within each window (Fig 5A, diagram). We then plotted these medians as a function of distance of the window from the cSSR. For sense transcripts, the median difference between WT and mutant log_2_(RPKM) value for genes within each window is very subtle, but is greater at closer distances to the cSSR (Fig 5B), indicating that the minor effect of base J and H3.V on sense transcription decreases with distance from the cSSR. When we examined antisense transcripts, we found that the deletion of base J and H3.V had a much greater effect (Fig 5C). In addition, this effect was distance dependent, with greater differences closer to the cSSR. The most pronounced effects were seen in *JΔ H3*.*VΔ* mutants (Fig 5C, blue line). We conclude that readthrough transcription in the absence of base J and H3.V results in the production of antisense transcripts in the neighboring PTU.

**Fig 6:**
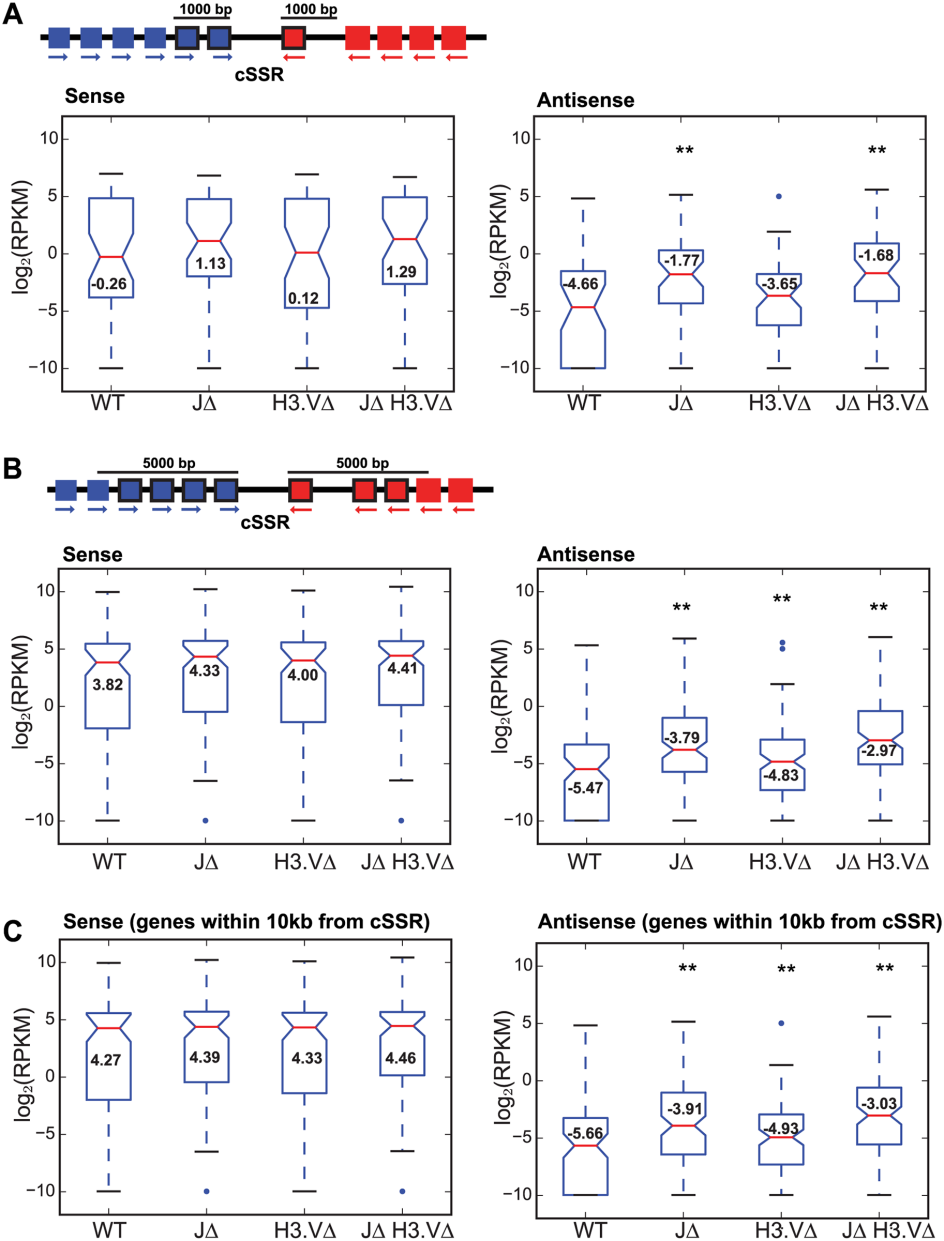
Deletion of base J and H3.V increases antisense transcription near convergent SSRs. (A) Top, schematic of computed values. log_2_(RPKM) values are calculated in WT or mutant cells for each gene that falls within 1,000 bp of a cSSR. In this example, 2 genes upstream of this cSSR and one gene downstream of this cSSR (indicated by solid black frames around the gene) would be included in the analysis. Bottom left, boxplot generated from log_2_(RPKM) values from sense transcripts of genes that flank cSSRs, defined as within 1,000 bp upstream of the 3´ end of the last gene in the (+) strand PTU or within 1,000 bp downstream of the 5´ end of the first gene in the (-) strand PTU in WT and mutant cells as indicated. Boxplots are displayed as in Fig 2G. Bottom right, same as bottom left except for antisense transcripts. ** indicates a significant difference (P < 0.01) between indicated mutant and WT values as measured by a Mann-Whitney U statistical test. (B) Top, same as diagram in A except the length has been extended to 5,000 bp so more genes are included in the analysis, in this case 4 genes upstream and 3 genes downstream of the cSSR. Bottom, boxplots were generated as in (Fig 3A) except that the length on either end of the cSSR has been increased to 5,000 bp. (C) Boxplots were generated as in (A) except that the length on either end of the cSSR has been increased to 10,000 bp.

As an extension of our analysis above, we asked whether transcription readthrough and the production of antisense transcripts might affect gene expression for those genes that are proximal to cSSRs. We used stranded polyA+ RNA-seq libraries generated from each of the mutants to address this question. We calculated log_2_(RPKM) values for all reads generated from both sense and antisense transcripts, and separately calculated log_2_(RPKM) values specifically from reads generated from sense strand transcripts or from antisense transcripts. A list of genes that are significantly up- or down- regulated by >4-fold, as measured by a T-test followed by Benjamimi and Hochberg correction in the mutants with associated p adjusted values is provided in S6 Table. Distances from the start of the gene to the nearest cSSR and to each flanking head-to-tail site is also provided in S6 Table. We first compared log_2_(RPKM) values for genes that fall within 1,000 bp of a cSSR (Fig 6A, top and S6A Fig). The results for sense reads are shown in the left-hand panels of Fig 6, and antisense reads in the right-hand panels. The results for all reads (both sense and antisense) are shown in S6 Fig. For each cSSR, we defined the 5´ interval as -1,000 bp from the end of the last (+) strand gene within the 5´ PTU, and the downstream interval as the first 1,000 bp from the start of the first (-) strand gene in the 3´ PTU. A gene was only included in the analysis if it fell within either interval. Boxplots comparing log_2_(RPKM) values for each genotype revealed an increase in the median log_2_(RPKM) for genes falling within 1,000 bp of the cSSR in each of the mutant lines for both sense and antisense reads, with the most pronounced increase in median log_2_(RPKM) found in the *JΔ H3*.*VΔ* mutant (Fig 6A). However, only the increase in antisense reads was statistically significant in both the *JΔ* mutant and the *JΔ H3*.*VΔ* mutant (S7 Table). Frequency distributions for the number of genes that fall within 1,000 bp upstream or downstream of a given cSSR reveal that at the majority of sites, 1–4 genes are within this range (S6A Fig, right). We conducted a similar analysis of genes that fell within 5,000 bp of the cSSR (Fig 6B, top diagram). For reads generated from sense transcripts, we observed only a subtle increase in median log_2_(RPKM) in each of the mutant lines, none of which were significant (Fig 6B, left panel, S7 Table). For reads generated from antisense transcripts, we observed an increase in the median log_2_(RPKM) value and a shift upward in the interquartile range for each of the mutant cell lines. The largest effect was once again seen in the *JΔ H3*.*VΔ* mutant cells (Fig 6B, right panel), but differences in transcript levels were also significant for the *JΔ* mutant when compared to WT transcript levels (S7 Table). For most sites, 1–8 genes lie within 5,000 bp of each cSSR (S6B Fig, right panel).

When we extended our analysis to 10,000 bp on either side of each cSSR, we found that the difference in median log_2_(RPKM) was again unaltered between the WT and the mutant lines for reads generated from sense transcripts (Fig 6C). However, expression levels for antisense transcripts were significantly higher in the *JΔ* and *JΔ H3*.*VΔ* mutants when compared to the WT, with the greatest effect again observed in the *JΔ H3*.*VΔ* mutant (S7 Table). These results indicate that deletion of both base J and H3.V does not change the level of sense transcripts for genes within 10kb of the cSSR. In contrast, the effect on antisense transcription of genes proximal to cSSRs appears to extend further out (up to at least 10,000bp) in the absence of both base J and H3.V (Fig 6C). A list of the genes analyzed for each distance window along with RPKM values is provided as S2 Data.

## Discussion

Here, we have investigated the effects of base J and H3.V in maintaining the transcriptional landscape surrounding their sites of deposition, which include cSSRs and telomeres. In general, we find that H3.V appears to be required for the maintenance of silencing of telomeric genes (*VSG*s), while both marks are important for proper transcription termination at the ends of PTUs. In the absence of these marks, the number of antisense transcripts following the end of a PTU is increased in the neighboring PTU. Previous work on the role for base J in pol II transcription termination was done using small RNA-seq on cells treated with dimethyloxalylglycine (DMOG) [13], and thus, in the context of pharmacologic inhibition of J production, these authors found no evidence for antisense transcription at cSSRs or at head-to-tail (HT) sites. However, they did detect changes in transcript levels for genes immediately downstream of some sites of J localization within PTUs in *JBP1Δ JPB2Δ* mutants using total RNA-seq. Our experiments on readthrough transcription were performed slightly differently, using polyA+ enriched libraries for genetic mutants. In contrast to previous results, we did find evidence for transcriptional readthrough at cSSR regions in *JΔ* mutant cells, which was exacerbated in the absence of H3.V (Figs 4 and 5). It is possible that the antisense transcripts that we detected might be processed by the cellular machinery in a way that does not lead to the production of small RNA degradation products, or that they are so short lived that they are undetectable, thus explaining why they were not detected using small RNA-seq. Alternatively, differences in the method for eliminating J (DMOG *vs*. genetic mutation) may have led to differences in the results from these two analyses. The total of number of genes where sense transcript abundance changes by > 2-fold in the *JΔ* is relatively low (total 62), most of these changes represent an increase in expression in the mutant, and many of the genes affected are *VSG*s, *ESAG*s, and RHS hotspots. This is in agreement with previous results using total RNA-seq on *JΔ* genetic mutants [13]. The number of transcripts whose abundance changes by > 2-fold in the *JΔ H3*.*VΔ* mutant is ~3 times higher than that of J alone, and ~13 times higher than *H3*.*VΔ* alone. However, these changes may be the result of secondary effects, since we did not find a specific pattern for changes in sense transcription immediately proximal to cSSRs.

Our data support a mechanism that requires the collaborative reading of histone variants as well as of a DNA modification in suppressing readthrough and antisense transcription at transcription boundaries, with the difference in severity of these transcriptional defects in *T*. *brucei* and *L*. *major* possibly due to a third chromatin mark that remains in BF *JΔ H3*.*VΔ* cells (perhaps H4.V or another, uncharacterized mark) to demarcate the ends of PTUs. At subtelomeres however, H3.V appears to be the dominant mark for suppressing transcription of inactive *VSG* genes by an unknown mechanism. Functional effects for the loss of base J and H3.V during infection remain to be studied in *T*. *brucei*, but studies in *T*. *cruzi* indicate that loss of *JBP1* and *JBP2* has functional effects on host cell invasion and egress [27]. Our findings that two independently deposited marks are coordinately read to affect transcription have relevance beyond *T*. *brucei* biology. For example, JBP1/2 are members of a larger family of α-ketoglutarate and Fe^2+^-dependent dioxygenases whose mammalian homologs are the ten-eleven translocation (TET) proteins (now referred to as the "TET/JBP family"; reviewed in [28]). TET proteins hydroxylate 5-mC to convert it to 5-hmC, but have also retained their ability to convert dT to 5-hmU [29]. A number of recent reports revealed functional links between TET-mediated DNA marks (hmC, hmU) and chromatin modifications (reviewed in [28,30,31]). For example, TET proteins can be attracted to particular loci on the basis of certain chromatin marks (e.g. promoter regions enriched with H3K4me^3^ and H3K27me^3^ mark [32]). In light of our work with H3.V and base J, we hypothesize that the binary reading of TET-mediated DNA marks together with a subset of chromatin marks results in the regulation of transcription close to termination sites. The transcriptional effects we observe in the homologs of these proteins in *T*. *brucei* might indicate that the DNA mark and histone mark, which do not depend on one another for deposition, might be read either by the same protein complex or by independent complexes that function within the same time frame. These complexes could potentially recruit different downstream factors to control repression at transcription boundaries and subtelomeres. Epigenetic marks and the proteins that modify or read them have been recognized as important factors in regulation of the expressed genome. Our study demonstrates a functional relationship between DNA and histone marks at transcription boundaries, and sheds light on the evolution of transcription regulation.

## Materials and Methods

### *Trypanosome* strains and plasmids used in this study

*Trypanosoma brucei* bloodstream forms (strain Lister 427) of the ‘single marker’ (SM) background expressing T7 RNA polymerase and Tet repressor (TetR) [33] were cultured in HMI-9 at 37°C [34]. Stable BF clones were obtained using electroporation (Lonza) and maintained in HMI-9 media containing antibiotics, as required, at the following concentrations: 2.5 μg/ml G418 (Sigma); 5 μg/ml blasticidin (Invivogen); 5 μg/ml hygromycin (Invivogen); 0.1 μg/ml puromycin (Sigma); 1 μg/ml phleomycin (Invivogen). The mutant cell lines and plasmids used for this study are listed in S8 and S9 Tables. Detailed construction information and maps are available upon request.

### Western blot of whole cells

Equal numbers of cells were collected and suspended in Laemmli buffer and separated on an SDS-PAGE gel. Following transfer to nitrocellulose, the proteins were analyzed using anti-VSG3 or anti-tubulin antibody.

### Switching assay by the Magnetic Activated Cell Sorting (MACS) and flow cytometry

Switching assays were performed as described previously [35,36]. Briefly, cells were maintained in the presence of blasticidin to exclude switchers from the starting population, as the blastidicin-resistance gene (*BSD*) was placed at the active BES promoter. Cells were then allowed to switch in the absence of selection for 3–4 days to ensure the same number of population doublings. Switchers were enriched using a MACS-column method. Flow-through enriched with switchers was collected and stained with anti-VSG2 antibody and propidium iodide (PI) for dead cell exclusion and analyzed by flow cytometry. Cells that lost VSG2 signal and are PI negative are live cells that have switched. Switching mechanism was assessed as described previously [36].

### RNA-seq

RNA was extracted from treated or control cells using RNA Stat-60 (Tel-Test) according to the manufacturer’s protocol and quantified on a NanoDrop2000c and further cleaned using RNase easy kit (Qiagen). Poly-A sequencing libraries were prepared by Rockefeller University Genomics Resources Center using the Illumina platform. Stranded sequencing libraries were prepared using the NEBNext Ultra Directional Library Kit (E7420). Sequencing was performed on an Illumina HiSeq 2000 sequencer using 50 bp reads. Reads were trimmed for quality using the TrimGalore program from Babraham Bioinformatics (http://www.bioinformatics.babraham.ac.uk/projects/trim_galore/) and aligned with Bowtie [37] to the reference genome (Tb927v5) allowing only for uniquely aligning reads with a maximum of 2 mismatches. Reads that did not align to the genome were then aligned to the Lister427 *VSG*nome (http://129.85.245.250/index.html) using the same parameters. Reads were quantified using SeqMonk (http://www.bioinformatics.babraham.ac.uk/projects/seqmonk) from Babraham Bioinformatics or, in the case of *VSG* aligned reads, custom python scripts.

For the plots shown in Fig 6, log_2_(RPKM) values for each gene were included in the analysis if they fell within a defined length of the cSSR. This length was defined as within 1,000 bp, 5,000 bp, or 10,000 bp upstream of the 3´ end of the last gene in the (+) strand PTU or within 1,000 bp, 5,000 bp or 10,000 bp downstream of the 5´ end of the first gene in the (-) strand PTU (see the diagrams in Fig 6). Genes falling within PTUs that were shorter than 1,000bp, 5,000bp, or 10,000bp were discarded from the analysis. For the plots in Fig 5, the distance of each gene from the cSSR was computed starting at the halfway point between the 3´ end of the last gene in the (+) strand PTU (for upstream genes) and 5´ end of the first gene in the (-) strand PTU (for downstream genes)(halfway point is demarcated by a black line in the Fig 5 diagram). Median difference in log_2_(RPKM) value between mutant and WT cells were computed for genes that fell within 5 kb windows proceeding upstream or downstream of the halfway point within the cSSR, defined as between the 3´ end of the last gene in the (+) strand PTU and the 5´ end of the first gene in the (-) strand PTU. For Fig 3, log_2_(RPKM) values were computed for the region that lies between the 3´ end of the last gene in the (+) strand PTU and the 5´ end of the first gene in the (-) strand PTU for each analyzed cSSR. For Fig 4, the maximal length 3´ UTR was defined using the data in [26]. The analysis was restricted to cSSRs 1–5 kb in length and flanked by genes with defined 3´ UTRs. For each cSSR, analysis was focused on three regions: (1) up to 1kb of the gene immediately upstream of the cSSR, (2) the cSSR itself, and (3) up to 1kb of the gene immediately downstream of the cSSR. For cSSR proximal genes shorter than 1kb, analysis was restricted to the length of the gene. Sliding windows were calculated by dividing each region into intervals 10% of the total length and sliding by a step size of 2%. For instance, 100 bp windows were slid 20 bp down the length of a 1 kb-long region. Median difference in log_2_(RPKM) values between mutant and WT cells were computed for windows in each cSSR and cSSR-flanking region. Median log_2_(RPKM) differences were then averaged for each window across all cSSR and cSSR-flanking regions. Notched boxplots were computed using python’s matplotlib library with the following command pylab.boxplot(data, notch = True, outliers = 'b.').

### Statistical analysis

Differences between WT and mutant cells were summarized using descriptive statistics performed in R, version 3.2.0 (The R Foundation for Statistical Computing). To compare *VSG* expression (Fig 2E, 2F and 2G), expression of cSSRs (Fig 3E), expression of cSSR proximal genes (Fig 6), and expression of HT sites (S5 Fig) a Kruskal-Wallis one-way analysis of variance test (command kruskal.test()) was first applied to determine whether a significant difference exists across median log_2_(RPKM) counts in WT and mutant cells. Once significance was established across groups, a Mann-Whitney U test (command wilcox.test()) was performed between median log_2_(RPKM) counts for each mutant genotype and the WT. Significance values in Figs 2E–2G, 3E and 6 correspond to the results of the Mann-Whitney U test. *P* < 0.05 was considered significant for all tests. For all graphs, *P* < 0.05 is indicated by * and *P* < 0.01 is indicated by **. Test statistics and *P* values are reported in S2, S4, S5 and S7 Tables.

### mRNA preparation and reverse-transcription-quantitative PCR

Total mRNA was prepared using RNA Stat-60 (Tel-Test) as described in manufacturer’s protocol and cDNAs were generated using random hexamer and reverse-transcriptase (Stratagene). RNA was amplified using primers specific to individual selected TTS regions by quantitative PCR using the LightCycler 480 (Roche). Amplified double-stranded DNA product during 40 cycles was detected by SYBR Green I. All measurements were in triplicate and compared with a 4,000-fold range of serially diluted standard genomic DNA prepared from the wild-type strain. The sequences of primers are available upon request.

### Stranded reverse-transcription-quantitative PCR

Stranded RT-PCR was performed as described above with the following differences. Gene specific primers were used to generate cDNAs from antisense or sense transcripts. These cDNAs were amplified using two sets of primers specific to the 5´ or 3´ region of each gene by quantitative PCR using the LightCycler 480 (Roche). Amplified double-stranded DNA products during 40 cycles were detected by SYBR Green I. All measurements were in quadruplicate and compared with a 16,000-fold range of serially diluted standard genomic DNA prepared from the WT strain. The sequences of primers are available upon request.

### Southern blot

To examine telomere growth, WT, *JΔ*, *H3*.*VΔ*, and *JΔ H3*.*VΔ* cells were transfected with pSY37, which targets the *HYG* selection marker right downstream of the active *VSG*2 and leaves about 200 bp telomere seed at the end of the chromosome. Over time, telomere seeds should elongate and elongation rate of telomeres was measured by Southern blot. About 200 million cells were collected and genomic DNA was prepared using QIAmp kit from Qiagen. Genomic DNA was digested with *Afl*II to measure the lengthening of BES-*VSG*2-telomere or with *Bgl*I to measure the length of silent BES-*VSG*3 telomere, and separated on an agarose gel. Specific *VSG*2 or *VSG*3 probes were used for detection.

## Supporting Information

S1 Fig Expression of silent VSG3 protein from an inactive BES is detected by western blot in the *H3*.*VΔ* and *JΔ H3*.*VΔ* trypanosome cells. Asterisk indicates non-specific band.
(EPS)

S2 Fig Base J and H3.V maintain silencing for a subset of *VSG* genes. Scatter plot showing log_2_(RPKM) values for all *VSG* genes for WT and indicated mutant cells. Dotted grey lines indicate changes that are 4-fold up or down. Comparisons between log_2_(RPKM) values WT and *JΔ* cells are show in (A), WT and *H3*.*VΔ* cells in (B), and WT and *JΔ H3*.*VΔ* cells in (C). (D). Notched boxplots of log_2_(RPKM) values were generated for all *VSG*s in WT and indicated mutant cells. Boxplots are displayed as in Fig 2G.
(EPS)

S3 Fig *VSG* switching and telomere length in base J and H3.V mutant cells. (A) Observed switching frequency in WT and indicated mutant cells. (B) and (C) Telomeres grow at a normal speed in all mutants. Diagram shows the targeting strategy. The plasmid pSY37 swaps the original telomere with the 200 bp telomere seed at the active *VSG*2 locus. Integration of the plasmid was confirmed by PCR and telomere length was analyzed by Southern blot using *VSG*2-specific probe. (D) and (E) Telomeres at the silent BES were maintained stably in all strains. Diagram shows the silent *VSG*3 BES and Southern blot probes. The probe also recognizes the 17.8 kb fragment (marked with asterisk).
(EPS)

S4 Fig Schematic of a chromosome portraying PTUs, convergent SSRs (cSSR), divergent SSRs (dSSR), location of base J, histones and histone modifications. HT, ‘head-to-tail’ site.
(EPS)

S5 Fig Transcription at HT sites. Notched boxplots of log_2_(RPKM) values were generated from stranded RNA-seq reads at HT sites in WT and indicated mutant cells. HT sites were defined as regions bound by Bdf3 that were not dSSRs. Boxplots are displayed as in Fig 2G. (A) Sense transcripts. (B) Antisense transcripts. (C) Both sense and antisense transcripts.
(EPS)

S6 Fig Transcript levels for genes close to cSSRs. (A) Top, schematic of computed values. log_2_(RPKM) values are calculated in WT or mutant cells for each gene that falls within 1,000 bp of a cSSR. In this example, 2 genes upstream of this cSSR and one gene downstream of this cSSR (indicated by solid black frames around the gene) would be included in the analysis. Bottom left, boxplot generated from log_2_(RPKM) values of genes that flank cSSRs, defined as within 1,000 bp upstream of the 3´ end of the last gene in the (+) strand PTU or within 1,000 bp downstream of the 5´ end of the first gene in the (-) strand PTU in WT and mutant cells as indicated. Boxplots are displayed as in Fig 2G. Bottom right, histogram plotting the frequency for the indicated number of genes that fall within 1,000 bp upstream of the 3´ end of the last gene in each (+) strand PTU (shown as negative values, yellow bars), or within 1,000 bp downstream of the 5´ end of the first gene in each (-) strand PTU (shown as positive values, blue bars) that flank convergent SSRs. (B) Top, same as diagram in A except the length has been extended to 5,000 bp so more genes are included in the analysis, in this case 4 genes upstream and 3 genes downstream of the cSSR. Bottom, boxplot and histogram were generated as in (A) except that the length on either end of the cSSR has been increased to 5,000 bp. (C) Boxplot and histogram were generated as in A except that the length on either end of the cSSR has been increased to 10,000 bp.
(EPS)

S1 Table Number of *VSG*s upregulated in indicated mutant strains with log_2_(RPKM)> -5. (DOCX)

S2 Table *P* values generated from Kruskal–Wallis one-way analysis of variance test across all genotypes and Mann-Whitney U test for transcript levels of *VSG*s in the indicated mutant strains compared to WT. *P* values < 0.05 indicated by * and *P* values < 0.01 indicated by **.
(XLSX)

S3 Table Number of TTS sites where transcript levels were significantly different (*P* value < 0.05) between replicate sets for indicated mutant cells compared to WT as measure by a T test using Benjamimi and Hochberg correction. (XLSX)

S4 Table *P* values generated from Kruskal–Wallis one-way analysis of variance test across all genotypes and Mann-Whitney U test for transcript levels of TTS sites in the indicated mutant strains compared to WT. *P* values < 0.05 indicated by * and *P* values < 0.01 indicated by **.
(XLSX)

S5 Table *P* values generated from Kruskal–Wallis one-way analysis of variance test across all genotypes and Mann-Whitney U test for transcript levels of HT sites in the indicated mutant strains compared to WT. *P* values < 0.05 indicated by * and *P* values < 0.01 indicated by **.
(XLSX)

S6 Table Number of genes where transcript levels were significantly different (*P* value < 0.05) between replicate sets for indicated mutant cells compared to WT as measure by a T test using Benjamimi and Hochberg correction. Only genes with fold-change >4 between mutant and WT cells are listed. Distance from the start of the gene to the nearest cSSR and to each adjacent head-to-tail site is also provided.
(XLSX)

S7 Table *P* values generated from Kruskal–Wallis one-way analysis of variance test across all genotypes and Mann-Whitney U test for transcript levels of genes proximal to cSSRs in the indicated mutant strains compared to WT. *P* values < 0.05 indicated by * and *P* values < 0.01 indicated by **.
(XLSX)

S8 Table *Trypanosoma brucei* strains used in this study. (DOCX)

S9 Table Plasmids used in this study. (DOCX)

S1 Data Excel file of raw counts of *VSG* reads. (XLSX)

S2 Data Excel file detailing the genes analyzed as proximal to cSSRs along with their RPKM values. (XLSX)
