# Supplementary material for: Base J and H3.V Regulate Transcriptional Termination in Trypanosoma brucei
Source: PLoS Genet. 2016 Jan 21;12(1):e1005762. doi: 10.1371/journal.pgen.1005762 (PMC4721952; doi:10.1371/journal.pgen.1005762)
Supplement: S1 Table — (DOCX) [file pgen.1005762.s007.docx]

**S1 Table.** Number of *VSG*s upregulated in indicated mutant strains with log_2_(RPKM)> -5.

| **Fold change** | ***VSG* type** | **Genotype** | **Number** |
| --- | --- | --- | --- |
| >4 | All *VSG*s | *J∆* | 45 |
|  |  | *H3.V∆* | 57 |
|  |  | *J∆ H3.V∆* | 193 |
|  | BES-*VSG*s | *J∆* | 0 |
|  |  | *H3.V∆* | 6 |
|  |  | *J∆ H3.V∆* | 7 |
|  | m*VSG*s | *J∆* | 0 |
|  |  | *H3.V∆* | 4 |
|  |  | *J∆ H3.V∆* | 5 |
|  | MC-*VSG*s | *J∆* | 2 |
|  |  | *H3.V∆* | 1 |
|  |  | *J∆ H3.V∆* | 13 |
