# Supplementary material for: Base J and H3.V Regulate Transcriptional Termination in Trypanosoma brucei
Source: PLoS Genet. 2016 Jan 21;12(1):e1005762. doi: 10.1371/journal.pgen.1005762 (PMC4721952; doi:10.1371/journal.pgen.1005762)
Supplement: S8 Table — (DOCX) [file pgen.1005762.s014.docx]

**S8 Table**. *Trypanosoma brucei* strains used in this study

| **Names** | **Life-cycle** | **Genotypes** | **Sources** |
| --- | --- | --- | --- |
| SM | BF | T7 RNA polymerase and Tet repressor (TetR)::*NEO* | [33] |
| HSTB-188 | BF | The BES1 promoter::*BSD* | [36] |
| HSTB-778 | BF | The BES1 promoter::*BSD,* pCRE-EP1  *JBP1∆/∆* *JBP2∆/∆* (=J∆) | This study |
| HSTB-826 | BF | The BES1 promoter::BSD  *H3.V∆PUR* / *H3.V∆HYG* (= *H3.V∆*) | This study |
| HSTB-827 | BF | The BES1 promoter::BSD  *H3.V∆PUR* / *H3.V∆HYG* (= *H3.V∆*) | This study |
| HSTB-828 | BF | The BES1 promoter::BSD  *H3.V∆PUR* / *H3.V∆HYG* (= *H3.V∆*) | This study |
| HSTB-829 | BF | The BES1 promoter::*BSD,*  pCRE-EP1  *J∆*, *H3.V∆PUR* / *H3.V∆HYG*  (= *J∆ H3.V∆*) | This study |
| HSTB-830 | BF | The BES1 promoter::*BSD,*  pCRE-EP1  *J∆*, *H3.V∆PUR* / *H3.V∆HYG*  (= *J∆ H3.V∆*) | This study |
| HSTB-831 | BF | The BES1 promoter::*BSD,*  pCRE-EP1  *J∆*, *H3.V∆PUR* / *H3.V∆HYG*  (= *J∆ H3.V∆*) | This study |
| HSTB-904 | BF | The BES1 promoer:: *BSD*, pCRE-EP1 | This study |
| HSTB-881 | BF | The BES1 promoter::*BSD,*  pCRE-EP1  *H3.V∆* | This study |
| HSTB-868 | BF | The BES1 promoter::*BSD,*  pCRE-EP1  *J∆ H3.V∆* | This study |
