# Supplementary material for: Base J and H3.V Regulate Transcriptional Termination in Trypanosoma brucei
Source: PLoS Genet. 2016 Jan 21;12(1):e1005762. doi: 10.1371/journal.pgen.1005762 (PMC4721952; doi:10.1371/journal.pgen.1005762)
Supplement: S9 Table — (DOCX) [file pgen.1005762.s015.docx]

**S9 Table**. Plasmids used in this study

| **Names** | **Inserts (markers) and targeting loci** | **Sources** |
| --- | --- | --- |
| pLEW100-  CRE-EP1 | Cre-recombinase inducible vector (*PHLEO*), rDNA spacer | [38] |
| pJEL38 | *H3.V* knock-out with *HYG* marker | [19] |
| pJEL76 | *H3.V* knock-out with *PUR* marker | [19] |
| pSY5 | *JBP1* knock-out with *HYG-TK* with loxP sites | This study |
| pSY14 | *JBP1* knock-out with *PUR-TK* with loxP sites | This study |
| pSY8 | *JBP2* knock-out with *HYG-TK* with loxP sites | This study |
| pSY15 | *JBP2* knock-out with *PUR-TK* with loxP sites |  |
| pDS37 | *JBP2* conditional knock-out with *PUR-TK* with loxP sites | This study |
| pDS88 | *H3.V* knock-out with *HYG-TK* with loxP sites | This study |
| pDS82 | *H3.V* knock-out with *PUR-TK* with loxP sites | This study |
| pSY37 | A vector targeting between *VSG2* and telomere with *HYG* marker | This study |

Reference

38. Scahill MD, Pastar I, Cross GAM (2008) CRE recombinase-based positive-negative selection systems for genetic manipulation in Trypanosoma brucei. Mol Biochem Parasitol 157: 73–82. doi:10.1016/j.molbiopara.2007.10.003.
